# Developing a Theory of Change and Implementation Plan to implement a novel child- and family-centred outcome measure in paediatric palliative care

**DOI:** 10.1177/02692163251331165

**Published:** 2025-04-17

**Authors:** Hannah May Scott, Inez Gaczkowska, Debbie Braybrook, Daney Harðardóttir, Lorna K Fraser, Clare Ellis-Smith, Richard Harding

**Affiliations:** Florence Nightingale Faculty of Nursing Midwifery and Palliative Care, Cicely Saunders Institute, King’s College London, London, UK

**Keywords:** Pediatrics, palliative care, children, implementation science, patient reported outcome measures, patient-centred outcomes research

## Abstract

**Background:**

To achieve benefits of person-centred outcome measures within routine children’s palliative care, implementation plans and the intended pathways to impact must be established.

**Aim:**

To develop a Theory of Change and Implementation Plan for sustained implementation of a novel person-centred outcome measure into routine hospital care for children with life-limiting conditions and to identify potential causal mechanisms.

**Design:**

Participatory workshops and a directed content analysis developed a Theory of Change. Framework analysis of workshop qualitative workshop data, supported by Normalisation Process Theory Informed an implementation plan.

**Setting/participants:**

Health and social care professionals and parent/carers were recruited to six online workshops through social media and networks.

**Results:**

Eight health and social care professionals and eight parents participated. The Theory of Change identified overall impact of improved care and quality of life, through improved identification of symptoms and concerns and improved communication between healthcare teams. However, for this to happen, education and training on the outcome measure, anticipated benefits, how to implement and use it are required. Logistical, resource and staffing barriers must be addressed, alongside the development of a detailed implementation plan. Analysis of workshop transcripts identified seven themes relating to the domains of Normalisation Process Theory: education and information needs, the importance of a tailored approach, stakeholder engagement and the role of champions, healthcare records and IT system support requirements, improved health outcomes, improved experience of care and evidence for service provision, development, evaluation and expansion

**Conclusion:**

Future work should pilot test the Theory of Change and Implementation Plan.

**What is already known on this topic?**

- Whilst person-centred outcome measures have been shown to improve the quality of care and patient outcomes in adult palliative care when successfully implemented into routine care, this has not been achieved for children and young people.
- It is essential to identify the causal mechanisms underpinning implementation and normalisation processes through which a novel child and family-centred outcome measure may achieve its intended impact, through the perspectives of stakeholders.

**What this paper adds?**

- A Theory of Change map was developed which explains the causal pathway for successful implementation of outcome measurement in children’s palliative care, including how short-term outcomes and long-term impacts may be achieved.
- The use of Normalisation Process Theory enabled novel understanding of implementation and normalisation of person-centred outcome measures into routine care.
- An implementation plan was developed to operationalise the strategies for implementation and normalisation of the C-POS:UK into routine care.

**Implications for practice, theory or policy**

- Theory of Change and Implementation Plan can be used to support the implementation of outcome measurement into routine care.
- Health and social care professionals and parents both described great potential for person-centred outcome measures to have a significant positive impact on health outcomes and experience of care for children with life-limiting conditions and their families.
- Future research should aim to assess the Theory of Change for transferability to support implementation and normalisation of person-centred outcome measures in routine care for children with life-limiting conditions in further contexts.

## Background

Children and young people (hereafter ‘children’) with life-limiting and life-threatening conditions (hereafter ‘life-limiting’) experience burdensome symptoms and concerns requiring a holistic, person-centred approach.^1,2^

Person-Centred Outcome Measures are standardised questionnaires that assess effects of a health condition or treatment on patients, and/or their family.^3^ They are usually self-completed or, when the patient is unable, proxy-completed.^3,4^ Their use can empower patients and families to raise concerns with clinicians, and support conversations and decision-making through shared language^5,6^; improving quality of care and outcomes.^7,8^ Despite a growing body of evidence on use and implementation of Person-Centred Outcome Measures in adult palliative care,^3,4,9–11^ evidence to underpin their use and implementation for children with life-limiting conditions is limited.^3,12–14^ Children with life-limiting conditions, their families and healthcare teams anticipate benefits of using such measures in care,^15^ including strengthening health and social care professionals’ (hereafter ‘professionals’) understanding of what matters to patients and families, improved communication and collaboration between families and healthcare teams, improved communication and collaboration between professionals and standardised data collection and reporting.^15^

The Children’s Palliative care Outcome Scale: UK version (C-POS:UK) project aims to develop,^1,16–23^ validate^23,24^ and implement^15,22,25,26^ a novel child- and family-centred outcome measure for use in the care of children with life-limiting conditions in the UK. However, to realise the benefits of C-POS:UK in care, implementation must be carefully considered,^9,11^ including the causal pathway,^9,11^ and mechanisms to sustained implementation and normalised use.

This study aimed to develop a Theory of Change and Implementation Plan for sustained implementation of C-POS:UK into routine hospital care for children with life-limiting conditions and to identify potential causal mechanisms.

## Methods

### Research questions

RQ1: What is the Theory of Change underpinning C-POS:UK implementation?
RQ2: What are the potential causal mechanisms underpinning sustained C-POS:UK use?

### Design

**Figure 1.**
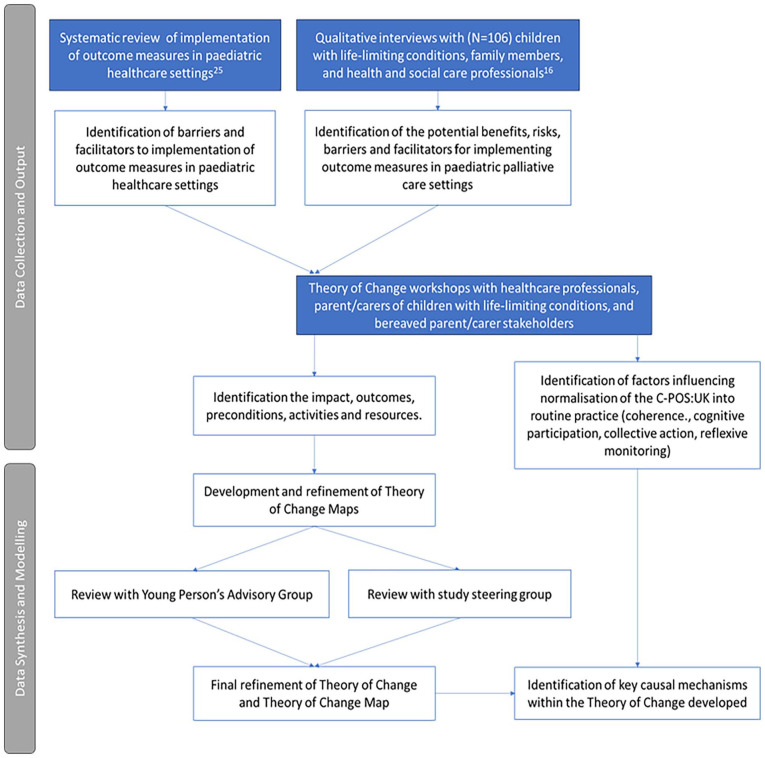
Diagram of information sources and synthesis process for developing the Theory of Change for the implementation of C-POS:UK.

This observational study used a participatory Theory of Change approach^27^ informed by the Medical Research Council Guidance for developing and evaluating complex interventions.^9^ Theory of Change workshops were conducted, informed by evidence from a systematic review on implementing Person-Centred Outcome Measures in paediatric healthcare^25^ and findings from interviews with stakeholders (including children with life-limiting conditions, their parents and siblings, health and social care professionals and planners of paediatric palliative care services).^15^ The prototype visual Theory of Change was reviewed in consultation with a Young Person’s Advisory Group and study steering group to form a final Theory of Change Map. To identify the key mechanisms underpinning sustained use, a second phase of qualitative analysis was conducted informed by Normalisation Process Theory.^28,29^ The methods for developing the Theory of Change are detailed in Figure 1.

The development of the Theory of Change is reported in line with Breuer et al.’s Theory of Change reporting checklist.^30^ Patient and Public Involvement with the Young Person’s Advisory Group is reported as per the Guidance for Reporting Involvement of Patients and the Public (GRIPP2) short checklist.^31^ The second phase of Framework analysis is reported in accordance with the consolidated criteria for reporting qualitative studies (COREQ).^32^

### Population

Inclusion criteria for participants are reported in Table 1.

**Table 1.** Inclusion criteria.

Participant type	Inclusion criteria
Health and social care professionals	Currently working in the UK National Health Service. At least 6 months professional experience caring for children (0–17 years) with life-limiting conditions.
Parents/carers (including siblings)	Aged 16 years or older. Has caring responsibilities for a child aged 0–17 years with any life-limiting condition.
Bereaved parents/carers (including siblings)	Aged 16 years or older who had caring responsibilities for a child with any life-limiting condition. At least 6 months post-bereavement to reduce the risk of distress.^33^

### Sampling and recruitment

Sampling was a combination of purposive (stakeholders targeted on the basis of being a parent/carer or bereaved parent/carer of a child with a life-limiting condition or being a professional working in the NHS caring for children with life-limiting conditions^34^) and convenience (social media as this is useful to access ‘hard to reach’ or ‘hidden’ populations in health research^35,36^).

Study advertisements (developed with the steering group) were disseminated through social media, mailing lists of a paediatric palliative care professional association and a family support non-governmental organisation, and through the steering group’s networks. Stakeholders who responded were screened for eligibility by HS.

### Data collection

Theory of Change workshops were held March–May 2023. Separate workshops were held for professionals, parents/carers and bereaved parents/carers to reduce the impact of power imbalances.^37^ Workshops for professionals were conducted asynchronously, on the online platform Basecamp^38^ providing discussion boards for participants to read and contribute in their own time.^39^ Workshops for parents/carers and bereaved parents/carers were conducted on MS Teams in small groups whereby participants and facilitators could see each other and interact in real time both verbally and through the chat function. Two workshops were held for each stakeholder group and participants could choose to attend one or both workshops.

The workshop topic guide was developed using findings from a systematic review^25^ and qualitative interview data^15^ in collaboration with bereaved parent Patient and Public Involvement study members. The workshops followed a structured format informed by STRiDE guidance^40^ with adaptations to support participation^37^ that is, shorter small group workshops to better accommodate parent/carers balancing caring responsibilities and to provide greater privacy. ‘Live’ delivery enabled the research team to provide real time support if needed. Workshops were asynchronous for professionals to maximise engagement alongside clinical demands.

The Theory of Change development used ‘backwards outcome mapping’, identifying the impact/long-term goals of implementing C-POS:UK then working backward to medium and shorter-term outcomes, preconditions and activities/resources that may be required.^40^ HS analysed the data to develop preliminary Theory of Change maps for each group which were presented for further discussion and refinement in the second workshops. The Young Person’s Advisory Group and steering group were consulted to further review the Theory of Change map and inform development of implementation strategies.

### Data analysis and integration

Workshop audio-recordings, and forum discussion boards were transcribed verbatim and pseudonymised, then analysed by HS using directed content analysis.^41^ A deductive coding framework was developed, informed by elements of Theory of Change.

The second phase of analysis applied a Framework analysis.^42^ Transcripts were inductively coded^42,43^ by HS and IG using NVivo 12. Interpretation was performed by HS by mapping the data to the domains of Normalisation Process Theory.^28,29^ Themes were then mapped onto the Theory of Change map and an Implementation Plan developed, reported in line with Proctor et al.’s recommendations for specifying and reporting implementation strategies.^44^

Findings were presented to the steering group (academics, researchers, advocates and professionals) to agree upon key strategies for the implementation plan. We also consulted a Young Person’s Advisory Group at a UK tertiary children’s hospital.^45^ Members were aged 13–24 years, with chronic health conditions, their siblings and young people interested in a career in healthcare or research. An age-adapted version of the Theory of Change map was developed for the consultation workshop. Members of the group were asked to provide feedback on the map content (what the most important elements were, if anything was missing).

### Ethical approvals and consent

Ethical approval was granted by King’s College London (HR/DP-22/23-33702). All participants provided written informed consent.

## Results

### Sample characteristics

Six workshops were conducted with a total of 16 participants. Demographic characteristics are reported in Table 2.

**Table 2.** Participant characteristics.

Parents/carers (*n* = 8)	*n* or mean (range)
Mean age (years)	48 (41–62)
Gender	
Female:male	6:2
Ethnicity	
White	7
Asian or Asian British	1
Relationship to child	
Mother	3
Bereaved mother	3
Bereaved father	2
Diagnosis of child (ICD 11 chapter headings)	
Metabolic	3
Neurological	2
Cancer	1
Neurodevelopmental	1
Perinatal	1
Mean age of child (years)	12 (8–22)
Health and social care professionals (*n* = 8)	*n* or mean (range)
Mean Age (years)	40 (35–46)
Gender	
Female:male	8:0
Profession	
Nurse	2
Lead nurse	2
Nurse specialist	2
Physiotherapist	1
Social worker	1
Years of experience (years)	16 (2–21)

### Theory of change for implementing C-POS:UK

**Figure 2.**
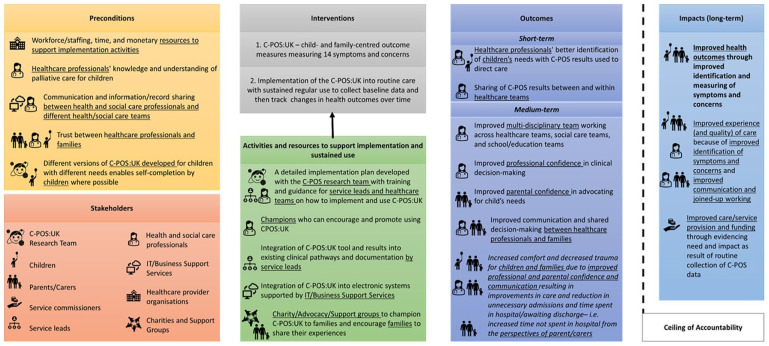
Theory of change map for implementation and routine use of the C-POS:UK into paediatric palliative care provided in UK National Health Service hospitals.

The Theory of Change map is presented in Figure 2.

### Impact

The anticipated long-term impact of implementing C-POS:UK was improved health outcomes due to better identification of symptoms and concerns. Professionals felt implementation of C-POS:UK would facilitate person-centred care and all participants reported that it could lead to improved experiences and quality of care. Professionals and parents suggested that the data collected from C-POS:UK could be used to evidence need and support service development/expansion at a commissioning level. Long-term impacts do however have a ceiling of accountability, due to other influencing factors.

### Outcomes

#### Short-term outcomes

Short-term outcomes included improved identification of children’s needs due to integration of into routine care, including sharing of C-POS:UK results between services. This, however, requires integration into existing clinical workflows and infrastructure.

#### Medium-term outcomes

C-POS:UK could provide professionals an understanding of what was important to families, improving shared decision-making. Parents felt it would support them advocating for their child’s needs and lead to improved collaboration with professionals. Parents felt that C-POS:UK could provide evidence of the child’s ‘baseline’ or ‘normal’ symptoms, giving professionals more confidence in making the decision to discharge, even with complex symptoms. This may reduce unnecessary time spent in hospital, leading to improved quality-of-life.

### Preconditions

Preconditions for successful implementation included adequate staffing, time, education/training needs and monetary resources. Parents reported that it is essential for all professionals (particularly those who are not paediatric palliative care specialists) to have an understanding of the speciality so that they have confidence to introduce and use the measure.

Electronic and paper-based versions of C-POS:UK are needed to accommodate differing needs and abilities of children, enabling them to self-report.

Parents felt that communication and information sharing between healthcare teams and services needed improvement before C-POS:UK was implemented (potentially though shared patient records), to avoid families having to re-complete C-POS:UK. A close, positive working relationship between families and professionals was identified as an important prerequisite.

### Activities and resources

Professionals recognised that implementing C-POS:UK would bring challenges but identified mitigating resources and activities. These included carefully planned clinical pathways, and a robustly developed implementation plan detailing families’ requirement that it is used flexibly in response to changing needs and family preferences.

Professionals felt incorporating C-POS:UK into existing tools (e.g. Child and Young Person Advanced Care Planning tools,^46^ Child and Family Wishes discussion tools^47^) and incorporating guidance into existing documentation may achieve seamless integration. Prompts and reminders when C-POS:UK is first implemented may also support integration. Professionals identified the need for IT system/business support to enable C-POS:UK to be integrated into electronic systems. Parents felt it would be important to involve charity/advocacy/support groups to help champion C-POS:UK with families, explain its benefits and encourage use.

### Review with study steering group

Twenty-four members attended the review meeting – 15 professionals (6 consultants, 7 nurses and 2 physiotherapists), 5 research team members and 4 clinical academics. To optimise implementation, attendees suggested the requirement to first get buy-in at an organisational level, then engage with service leads, then individual professionals and families. Attendees also recommended champions to encourage and promote C-POS:UK use. Training and education on how to implement and use C-POS:UK in practice was also identified as important, alongside ensuring C-POS:UK can be integrated into existing clinical pathways and systems.

### Consultation with a Young Persons Advisory Group

Thirty-one young people (19 female and 12 male) aged 13–24 years attended an online meeting in July 2023. All members reported that the Theory of Change map was comprehensive. However, they stressed that C-POS:UK should be implemented and used flexibly to best enable children to self-report. Members also proposed that sharing of C-POS:UK data should go beyond the hospital setting to include other healthcare teams and teaching/education providers in the community.

### Framework analysis main findings

**Figure 3.**
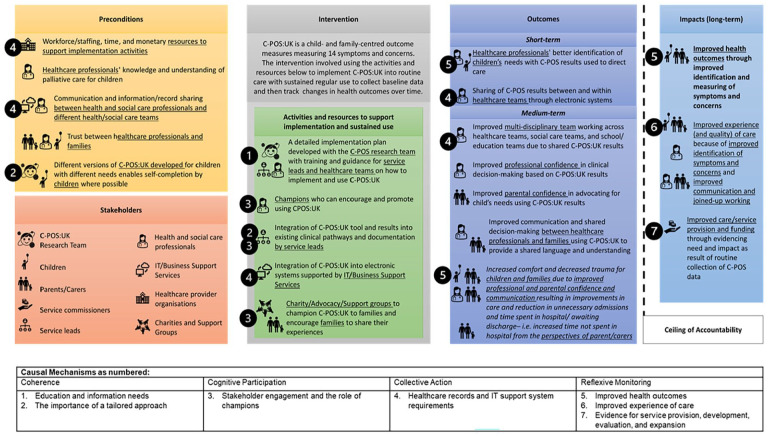
Theory of change map for implementing the C-POS:UK into routine paediatric palliative care within hospitals with key causal mechanisms underpinning implementation and normalisation mapped.

Seven themes were identified across the four domains of Normalisation Process Theory: coherence, cognitive participation, collective action and reflexive monitoring to sustain implementation. The themes are described in the text below and Figure 3 shows themes mapped onto the Theory of Change.

### Coherence

#### Theme 1: Education and information needs

All participants discussed education and information needs. Professionals’ needs related to practical aspects, for example, understanding fit within existing clinical workflows and when/how it should be completed. Professionals proposed providing a plan for initial implementation and guidance for incorporation into existing documentation. Family needs related to understanding what C-POS:UK is and benefits of use.

*All stakeholders need to know the purpose and importance of the C-POS. Clinicians and families need to know how to complete the C-POS. Clinicians need to know how to record the [completed C-POS results] on the electronic patient record. –* HSCP10

*Implementing change is always tricky but if a clear clinical pathway was implemented on how to do this it would be less challenging. –* HSCP12

*I think here it’s all about the why, so why are you asking people and what benefit is it going to have and I think that’s that needs to be really dealt with. –* PARCAR8

#### Theme 2: Importance of a tailored approach

Professionals felt C-POS:UK could be used routinely during existing review meetings to support better identification of changing needs.

*Ideally it would be used on a semi-regular basis (during annual reviews or other important meetings) in order to direct care appropriately in relation to changing outcomes. –* HSCP9

However, both professionals and parents emphasised C-POS:UK should be used flexibly due to changing needs, with more frequent use if a child became unstable.

*As we all know, each family is different [. . .] Mandating a frequency for completion is unlikely to be in keeping with the preferences of families and may actually be detrimental to their outcomes. –* HSCP9

Some parents felt there was a specific professional they had a close relationship with that may be most appropriate to compete C-POS:UK with. The professional may be different for each family and may change over time.

*for us that was definitely who we saw the most of erm and a real relationship was built up [. . .] because they were seeing you on a weekly basis whereas consultants you know could be every six months perhaps three but it’s over many years you do build a good relationship but it is a less week-on-week thing erm but as I say it that does seem to work differently everywhere in the country –* PARCAR6

Parents and professionals also discussed the importance of having electronic and paper-based versions of C-POS:UK, for children with different needs or abilities.

*and different ways of completing it you know [. . .] because he’s deaf but he can hear some stuff with something that was visual that he was able to rate or he could do it on an iPad.* – PARCAR1

### Cognitive participation

#### Theme 3: Stakeholder engagement and the role of champions

Professionals and parents highlighted the importance of engaging stakeholder groups and proposed identifying champions. Champions would likely be professionals who could drive implementation, educating and training other professionals on how C-POS:UK may support care provision, demonstrating its use with patients and families and providing prompts/reminders.

*I think that by having professionals who are passionate and competent about the benefits of using the [outcome measure] to educate and train teams in use of it should help to address any barriers of reluctance in incorporating it into the core tools used with this service user group. –* HSCP4

For parents, understanding why they were being asked to complete more paperwork was felt to be key to motivating families. Hearing the benefits from other parents and through family support groups were felt to be effective ways to develop a community around its use.

*if they have another parent saying to them use this it is a lifesaver they will go for it [. . .] how it works and why it works well and sort of share our experiences about it –* PARCAR9

### Collective action

#### Theme 4: Healthcare records and IT support system requirements

Parents felt that C-POS:UK results could be kept as part of healthcare records or passports to facilitate sharing. However, professionals noted where services were not all using the same digital systems, this may complicate sharing of C-POS:UK results.

*her folder had her symptom management plan, advanced care plan, um her standard care plan and her [Medication Administration Record] charts for all the different drugs she took and er escalation protocols for anything like dystonic episodes, etc. etc. It was all there in one place. Um so a tool like this in there would’ve been fantastic as well. –* PARCAR3

*Having shared records that all professionals that have consent to access can access as some providers are not even digital, communication is the key to everything being successful –* HSCP12

Professionals recognised potential challenges to integrate C-POS:UK into online infrastructure, and the need for IT system support

*Anything that requires the developers of the electronic patient record to make changes at the developers’ side [may be a challenge]. [. . .] We have identified an issue with another outcome measure which requires the [IT system] developer to make a change at their end; we have been asking for the change for several years. –* HSCP10

### Reflexive monitoring

#### Theme 5: Improved health outcomes

Professionals and parents felt implementation and regular use of C-POS:UK in care would support better identification and management of symptoms and concerns, leading to improved outcomes.

*The ultimate goal would be to improve health outcomes of care by identifying what will make a positive impact on our patients and their families. –* HSCP2

*hopefully their wellbeing or comfort will have increased as a result of there being some measurable data to assess the situation against –* PARCAR2

#### Theme 6: Improved experience of care

Professionals expected that C-POS:UK use would improve experiences of care by supporting a person-centred approach.

*The measure will help us to identify outcomes that are important to children/young people and their families and working to improve those outcomes will almost certainly improve their care experience –* HSCP9

Improved care experiences may be achieved through a shared understanding of what was most important to improve shared decision-making. Parents described how having data from C-POS:UK would support them to advocate for their child’s needs, and lead to greater collaboration between families and professionals.

*yeah I think what this would do this would encourage that collaborative approach in care from the parent carers and from the clinicians which they all strive to do and in theory it should breakdown those barriers so there’s going to be no unnecessary confrontations which I think is going to be really cool actually* – PARCAR8

#### Theme 7: Evidence for service provision, development, evaluation and expansion

The collection of routine, standardised data that was also seen as beneficial by both parents and professionals. Parents felt the availability of data would provide evidence of need and better enable children and families to access services and support.

*I wonder if that connected or produce reports they could use, because I remember when we finally said yes we want to have carers at home at night because we are not sleep. It was a real big headache to get it all together and it was all the actual medical information and we again had to resort to our known nurses and the people that really know us that say that they’re exhausted and really need this help and [child’s] condition is really really really severe – PARCAR9*

Professionals recognised the potential impact at a service or system level, as data could provide evidence of need to help direct development or expansion of services.

*If effective the implementation could provide evidence for service development and expansion to ensure outcomes improve for patients and families –* HSCP7

## Implementation plan

From the Theory of Change workshops, review with the Young Person’s Advisory Group and study steering group, and analysis of the workshop transcripts, an implementation plan was developed. It is formed of strategies to achieve the potential key causal mechanisms identified. The plan was developed using Normalisation Process Theory to ensure both initial implementation and sustained use. The strategies reported in Table 3 can be used alongside the Theory of Change map to support implementation. To ensure greater specification and operationalisation, the strategies are reported in line with Proctor et al.’s recommendations for specifying and reporting implementation strategies^44^ and drawing on Powell et al.’s compilation to ensure clarity and consistency in the naming of the strategies.^48^

**Table 3.** Implementation plan for Integration of C-POS:UK into routine paediatric palliative care with specification of implementation strategies.

Domains	Strategy: develop a formalised implementation blueprint and mandate change
Actor(s)	Intervention developers, patient/family/healthcare professional stakeholders, Implementation/Service Leads or Champions
Actions(s)	This plan consisting of several strategies has been developed alongside key stakeholder groups. Implementation/Service Leads or champions must mandate change and declare the C-POS:UK is being implemented within the care they provide. C-POS:UK should be completed by a known/trusted healthcare professional with the child and family (prioritising child self-report where possible) at a timeframe that is agreed upon by the family during routine visits or appointments based on the child and family’s need (for children who are more stable this may be around every 3–6 months and for children who are unstable or deteriorating this may be around every 1–3 weeks). Based on the C-POS:UK answers the healthcare professional should discuss next steps with the child and family and record these in the patient’s record, where possible, families should be provided a copy of the completed C-POS:UK. The C-POS:UK results and care plan should be discussed with the healthcare team and actioned and a timeframe for repeating completion of the C-POS:UK agreed upon. This should happen each time a C-POS is completed so children and families can see the changes in outcomes and are more involved in the shared decision-making process.
Target(s) of the action	Healthcare professionals understand how and when to use the C-POS:UK.
Temporality	The implementation plan has been developed prior to implementation. The implementation of the C-POS:UK should be mandated at the beginning of implementation and use in routine care.
Dose	C-POS:UK should be completed on a timeframe that is agreed upon by the family during routine visits or appointments based on the child and family’s need (for children who are more stable this may be around every 3–6 months and for children who are unstable or deteriorating this may be around every 1–3 weeks) and discussed weekly at multi-disciplinary team meetings.
Implementation outcome(s) affected^49^	A formalised implementation blueprint with a clear clinical pathways for what C-POS:UK use should look like should ensure C-POS:UK is more likely to be used as intended (fidelity).
Justification (research or theory)	Qualitative interviews^15^ Theory of Change workshops Secondary Frameworks analysis
Domains	Strategy: Change record systems and documentation
Actor(s)	Service leads or IT/Business support staff
Actions(s)	Record systems must be updated to integrate C-POS:UK into the electronic systems and enable prompting of the measure for eligible patients and to ensure it can be recorded appropriately. Documentation and clinical guidance must be updated for example, to include C-POS:UK in annual reviews.
Target(s) of the action	Healthcare professionals will have easier access and ability to use and record the C-POS:UK within the care they provide
Temporality	Changes must be made ahead of C-POS:UK being used in care
Dose	This should occur once at the beginning of implementation and reviewed regularly when systems or documentation changes.
Implementation outcome(s) affected^49^	If C-POS:UK is integrated into existing systems and documents, this will improve penetration. This in turn should also improve perceived feasibility of using it and reduce changes of disruption to existing workflows increasing adoption and improving the likelihood C-POS:UK will be used as intended (fidelity). This should increase the acceptability of C-POS:UK to healthcare professionals and increase chances of sustainability over time.
Justification (research or theory)	Systematic Review^25^ Qualitative interviews^15^ Theory of Change workshops Secondary Frameworks analysis
Domains	Strategy: develop effective education materials, conduct educational meetings and distribute educational materials
Actor(s)	Intervention developers in collaboration with key stakeholder groups (patients/family members and healthcare professionals)
Actions(s)	Education/Training materials should be developed and produced by intervention developers in collaboration with key stakeholders. These should include: • education/training workshops for healthcare professionals explaining what C-POS is, what the benefits are and how to implement and use it in care, • short overview videos (1–2 min) so all healthcare professional caring for children with life-limiting conditions staff are aware of what C-POS is and why it is being used,^50^ • longer (approximately 5 min) more detailed videos on how to use C-POS:UK in practice for healthcare professionals completing the C-POS:UK with families, • short videos (1–2 min) for children, young people and families that briefly explain what C-POS:UK is and why they are being asked to complete it,^51^ • booklets/leaflets for children and families that briefly explain what C-POS:UK is and why they are being asked to complete it.
Target(s) of the action	Healthcare professionals’ knowledge of C-POS:UK will be increased including how to implement and use in care and attitudes towards C-POS:UK improved due to knowledge of the benefits over current practice. Children and family’s knowledge of the C-POS:UK will be increased and attitudes towards C-POS:UK improved due to knowledge of the benefits.
Temporality	Workshops for healthcare professionals should be held at the beginning of implementation. Other educational materials should be distributed and disseminated from initial implementation onwards
Dose	Initial workshops should be held once at the beginning of implementing C-POS:UK within a healthcare setting for all healthcare professionals involved. Materials will be distributed on an ongoing basis.
Implementation outcome(s) affected^49^	Training and educational materials on how to implement and use C-POS:UK for healthcare professionals should improve initial adoption, fidelity and penetration. Knowledge of the benefits of the C-POS:UK should increase perceived acceptability for healthcare professionals, children and families.
Justification (research or theory)	Systematic review^25^ Qualitative interviews^15^ Theory of Change workshops Secondary Frameworks analysis
Domains	Strategy: identify and prepare champions/implementation leads
Actor(s)	Healthcare professionals, team/service leads, intervention developers
Actions(s)	Team/service leads identify individuals or individual healthcare professionals identify themselves to act as implementation leads or champions. Intervention developers provide additional training and support to prepare the implementation leads/champions to drive implementation in their setting. Implementation leads/champions can answer questions and provide encouragement and reminders to use the C-POS:UK.
Target(s) of the action	Implementation leads/champions are identified and gain additional knowledge to support implementation. Healthcare teams are encouraged and reminded to use the C-POS:UK routinely.
Temporality	Implementation leads/champions should first be identified prior to initial implementation to allow time for training. Additional champions can be identified once C-POS:UK is implemented
Dose	This work should be ongoing.
Implementation outcome(s) affected^49^	Implementation leads and champions support greater initial adoption and use of C-POS:UK by healthcare team members. As they can prompt and encourage use, this should lead to improved fidelity short-terms and greater penetration and sustainability in the long-term.
Justification (research or theory)	Systematic review^25^ Theory of Change workshops Secondary Frameworks analysis
Domains	Strategy: Facilitate relay of clinical data to providers
Actor(s)	Key workers or administering healthcare professional
Actions(s)	Once C-POS:UK has been completed with child and family and a care plan agreed upon, the completed C-POS:UK and care plan should be shared with all healthcare professionals/teams/services that are supporting the child and family (where relevant), for example, hospice, community nurses
Target(s) of the action	Improved communication and collaborative working across and within healthcare teams as all professionals understand the current unaddressed needs of the child and family and can support with the care plan.
Temporality	As soon as first C-POS:UK is completed by child and family.
Dose	After every C-POS:UK completion
Implementation outcome(s) affected^49^	The additional workload and new process may reduce acceptability. However sharing of C-POS:UK results may save costs and time of conducting similar assessments across multiple services supporting the family in the longer term. Sharing across services/teams may also increase C-POS:UK penetration.
Justification (research or theory)	Systematic review^25^ Qualitative interviews^15^ Theory of Change workshops Secondary Frameworks analysis
Domains	Strategy: Prepare patients and family members to be active participants, and obtain patient and family feedback
Actor(s)	Intervention developers and patient advocacy/family support groups/charities
Actions(s)	Intervention developers should work with patient advocacy/family support groups/charities to promote to C-POS:UK and it’s benefits to the patients and families they support, and encourage them to share their experiences with other patients and families
Target(s) of the action	Patient and family knowledge of and motivation to use the C-POS:UK
Temporality	Once C-POS:UK has been validated and is being used in clinical practice.
Dose	This work should be ongoing
Implementation outcome(s) affected^49^	C-POS:UK intervention is viewed as more acceptable by patients and families. Uptake (adoption) of C-POS:UK by patients and families in clinical care is increased. Feedback from patients and families may also improve sustainability.
Justification (research or theory)	Theory of Change workshops Secondary Frameworks analysis

Specification adapted from Proctor et al.^44^

## Discussion

### Main findings

Through using a Theory of Change approach, a causal pathway for the implementation of the novel C-POS:UK was developed and presented in a Theory of Change Map. Utilising Normalisation Process Theory to interpret the data enabled novel understanding of potential normalisation of C-POS:UK into sustained routine care. From this work an implementation plan was developed consisting of strategies to operationalise future implementation efforts.

The professional perspective enabled identification of important service/system level factors such as electronic system support and integration. The parent perspective prioritised individual/family level factors such as flexible use of C-POS:UK to accommodate changing needs throughout the child’s life.

The review with the steering group and consultation with the Young Person’s Advisory Group highlighted key elements of the Theory of Change that must be considered when C-POS:UK is implemented into routine care.

### What this study adds?

Whilst many of the findings are in keeping with both the existing adult palliative care literature and findings from other paediatric care settings,^3,7,8,25,52,53^ this is the first time they have been identified in the context of paediatric palliative care. Considerations that are specific to this population and context relate to the importance of ensuring C-POS:UK is implemented in a way that enables children to self-report their own health outcomes and that it equally engages both the child and their family members. This must be carefully considered in relation to delivery mode, as well as how the results are shared and stored across services, including issues relating to data privacy.^16^

As with all complex interventions, the implementation of the C-POS:UK is context-specific, and this Theory of Change has been developed for a specific healthcare context – paediatric palliative care provided in UK National Health Service hospitals. We recommend a similar method (i.e. Theory of Change) is applied in further care delivery settings such as community and hospice.

As far as we are aware, this is the first time that Theory of Change workshops have been conducted through multiple small group workshops and using a mix of synchronous and asynchronous methods. During the time-period in which the workshops were run, there were multiple strains on paediatric healthcare services in the UK including a scarlet fever outbreak and nursing and junior doctor strikes. This led us to take the decision to take an asynchronous approach to the professional workshops. This approach allowed professionals to engage in the workshops at a time that would work best for them.

For parents/carers, we ran synchronous workshops at different times and days (without the requirement to attend all workshops) which provided parents/carers greater opportunity to participate. The small groups also provided more privacy for parents/carers to share their experiences, compared to the larger groups more typical of traditional Theory of Change workshops. The small groups also enabled the research team to probe participants’ answers and suggestions, to better understand their thoughts and reasoning. This novel approach has advanced Theory of Change methodology, providing evidence that different modes of engagement can produce robust Theory of Change development and enable engagement with stakeholder groups who may otherwise have not been able to participate.

Whilst several methods for selecting implementation strategies have been proposed,^54^ they typically rely on theory and/or existing research. However, there is very limited existing research into implementing an outcome measure into paediatric palliative care.^15,25^ As such this study took a stakeholder-driven approach, to identify key considerations for implementation that were developed into strategies. We found this approach feasible and our systematic reporting, could enable this approach to be replicated in future research to develop implementation strategies where there is limited evidence.

### Strengths and limitations

The sample is a potential limitation of this study. Only a quarter of the parent/carer participants were fathers. This is in line with much of the existing paediatric palliative care research, where fathers are often underrepresented (mothers made up 75% of samples across 45 studies).^55^ Additionally, the majority of the parent/carers were white British. This is a limitation as in the UK life-limiting conditions are more prevalent in children from Pakistani, Asian and Black backgrounds.^56^ As such, there may be additional implementation considerations specific to those from ethnically diverse backgrounds.^57^ Further work is required to meaningfully engage and involve children with life-limiting conditions and their family members from ethnically diverse backgrounds.^58,59^ Innovative child-friendly approaches may also need to be developed to further enable the participation of children.^60,61^ Reduced ability to probe in the asynchronous workshops was a limitation of the professional workshops meaning there may be additional considerations that could impact implementation that have not been identified.

Finally, due to lack of existing measures in practice,^62^ implementation of C-POS:UK was presented in a hypothetical way to stakeholders. This work provides a theoretically informed explanation of how implementation of C-POS:UK may occur. Further work is needed to test the proposed causal pathways of the Theory of Change and Implementation Plan in practice through pilot and/or feasibility studies including addressing resource/logistical challenges.^15,25^

## Conclusion

Using a participatory Theory of Change approach, a potential causal pathway and key mechanisms have been identified for implementation and normalisation of the novel C-POS:UK into hospital-based paediatric palliative care and an implementation plan has been developed. There is great potential for C-POS:UK to have a significant positive impact on the care provided to children with life-limiting conditions and their families.
